# Adaptive Immune Responses in Human Atherosclerosis

**DOI:** 10.3390/ijms21239322

**Published:** 2020-12-07

**Authors:** Silvia Lee, Benjamin Bartlett, Girish Dwivedi

**Affiliations:** 1 Department of Advanced Clinical and Translational Cardiovascular Imaging, Harry Perkins Institute of Medical Research, Murdoch 6150, Australia; benjamin.bartlett@research.uwa.edu.au (B.B.); girish.dwivedi@perkins.uwa.edu.au (G.D.); 2 Department of Microbiology, Pathwest Laboratory Medicine, Murdoch 6150, Australia; 3 School of Medicine, University of Western Australia, Nedlands 6009, Australia; 4 Department of Cardiology, Fiona Stanley Hospital, Murdoch 6150, Australia

**Keywords:** human atherosclerosis, adaptive immune response, T-cells, B-cells

## Abstract

Atherosclerosis is a chronic inflammatory disease that is initiated by the deposition and accumulation of low-density lipoproteins in the artery wall. In this review, we will discuss the role of T- and B-cells in human plaques at different stages of atherosclerosis and the utility of profiling circulating immune cells to monitor atherosclerosis progression. Evidence supports a proatherogenic role for intraplaque T helper type 1 (Th1) cells, CD4^+^CD28^null^ T-cells, and natural killer T-cells, whereas Th2 cells and regulatory T-cells (Treg) have an atheroprotective role. Several studies indicate that intraplaque T-cells are activated upon recognition of endogenous antigens including heat shock protein 60 and oxidized low-density lipoprotein, but antigens derived from pathogens can also trigger T-cell proliferation and cytokine production. Future studies are needed to assess whether circulating cellular biomarkers can improve identification of vulnerable lesions so that effective intervention can be implemented before clinical manifestations are apparent.

## 1. Introduction

Atherosclerosis is a chronic vascular disease involving endothelial dysfunction following the deposition and accumulation of lipoproteins (e.g., low-density lipoproteins (LDL)) in the arterial intima. Plaque rupture and subsequent arterial occlusion can lead to myocardial infarction and stroke, which are the leading causes of death worldwide. The initiation and progression of atherosclerosis involve inflammatory pathways, with the recruitment and activation of several immune cell types and the release of soluble mediators [1]. Cells of the innate immune system, primarily macrophages, can take up modified oxidized LDL (oxLDL) and transform into foam cells to form fatty streaks (“early plaques”) [2]. Further lipid accumulation and leukocyte infiltration into established atherosclerotic plaques (“advanced”) create a core region with a collagen-rich fibrous cap established by vascular smooth muscle cells [2]. Plaques can be categorized into two broad categories: stable or unstable. Stable plaques are characterized by a small lipid core, few inflammatory immune cells, and a thick fibrous cap. Unstable (also known as “vulnerable”) plaques often have a very large lipid core, a thin fibrous cap, and intraplaque hemorrhage [2].

Cells of the innate and adaptive immune system are present in the different layers (intima, media, and adventitia) of the artery walls throughout the development of atherosclerotic plaques in mice and humans [3,4]. Studies using gene knockout mice have enabled detailed analyses of immune cells involved in atherogenesis [4], whilst human studies are more limited. However, it is important to highlight that different genetic and environmental factors affect atherogenesis in mice and humans. It is well established that different subsets of monocytes and macrophages play a crucial role in the atherosclerotic plaque establishment and disease progression [4]. In this review, we will summarize the roles of adaptive immune cells in human atherosclerotic plaques and review the clinical utility of profiling circulating immune cells in this context.

## 2. Development of T-Cell Subsets

**Figure 1:**
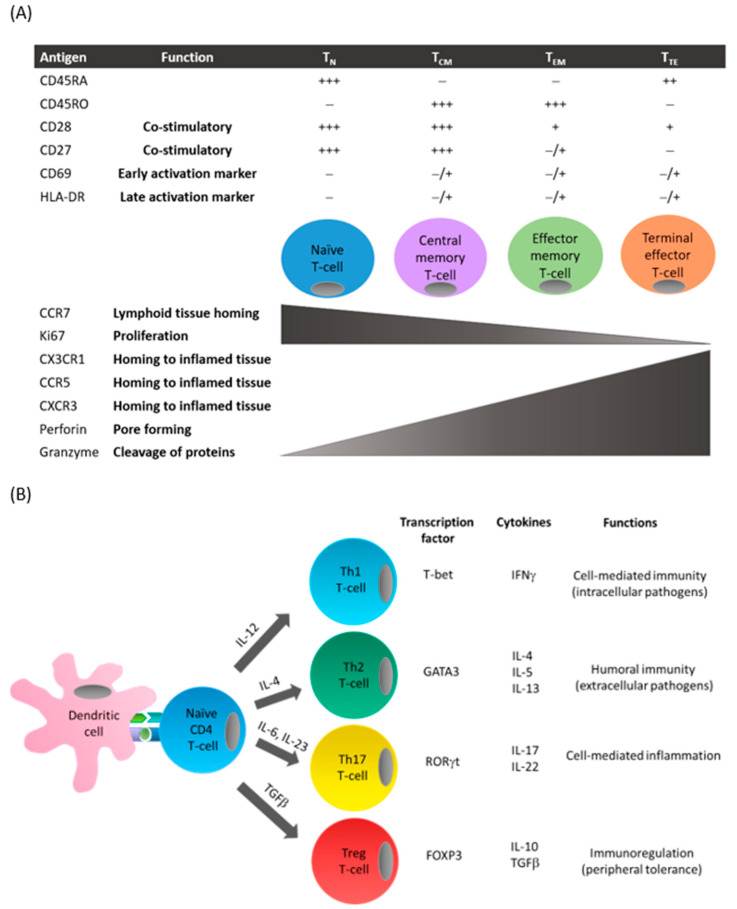
T-cell differentiation. (**A**) Following antigen presentation by antigen-presenting cells such as dendritic cells, naïve T-cells can differentiate to central memory or effector T-cells. This process is associated with the presence (+) or absence (−) of cell surface receptor expression on T-cells including costimulatory molecules and chemokine receptors, and functions including proliferation and cytotoxicity. (**B**) Depending on the costimulatory signals and the cytokines produced by antigen-presenting cells in the surrounding microenvironment, CD4^+^ T-cells express specific transcription factors that favor the differentiation into the different T-cell subsets. These subsets can be characterized by their distinctive cytokine secretion profile and associated effector functions.

Following antigen presentation by antigen-presenting cells (e.g., dendritic cells) and recognition by T-cell receptors, naive CD4^+^ and CD8^+^ T-cells (T_N_) differentiate into central memory (T_CM_) or effector memory T-cells (T_EM_) [5] (Figure 1A). T_CM_ generally reside in lymphoid tissues and are primed for rapid response to a previously encountered antigen. Following activation, T_CM_ undergo clonal expansion and differentiate into T_EM_. T_EM_ can be defined by their expression of the cell surface receptor, CD45RO, and low levels of costimulatory receptors (e.g., CD28). Furthermore, these cells display loss of chemokine receptors (e.g., CCR7) necessary for cells to migrate to secondary lymphoid tissues such as lymph nodes and spleen, whilst there is upregulation of receptors required for migration to peripheral sites of inflammation (e.g., CCR5 and CXCR3). In response to antigens, both T_CM_ and T_EM_ can produce effector cytokines, but T_CM_ possess higher proliferative capacities. Chronic exposure to antigens results in the generation of terminal effector T-cells (T_TE_) that proliferate poorly but have strong cytokine-producing and cytotoxic capabilities.

Depending on the antigen, costimulatory signals, and cytokine milieu of the microenvironment, naïve CD4^+^ T-cells differentiate into distinct T helper (Th) lineages (Figure 1B). T helper 1 (Th1) cells produce predominantly interferon (IFN)γ, Th2 cells produce interleukin (IL)-4, IL-5, and IL-13, and Th17 cells secrete IL-17 and IL-22 [5]. Differentiation into Th1, Th2, or Th17 subsets is controlled by specific transcription factors: T-bet, GATA3, and RORγt, respectively. Each Th cell subset possesses a unique expression profile and specific functions during an immune response.

Macrophages play a crucial role in regulating Th1/Th2 immune responses and can be broadly classified into two main subtypes: classically activated M1 macrophages and alternatively activated M2 macrophages [4]. M1 macrophages direct T-cells to produce Th1 cytokines to stimulate cytolytic activity and recruit more M1 macrophages and are considered proinflammatory. In contrast, M2 macrophages stimulate T-cells to produce Th2 cytokines to induce B-cell proliferation and amplify M2 macrophage responses and are considered anti-inflammatory. An imbalance of macrophage M1-M2 polarization can lead to inflammation and disease.

Human regulatory T-cells (Treg) can suppress the activation of immune cells through cell-to-cell contact and/or the secretion of inhibitory cytokines (e.g., transforming growth factor (TGF)-β and IL-10) [6]. They are characterized by the expression of the transcription factor forkhead box P3 (FOXP3) and the IL-2 receptor α subunit, CD25.

## 3. T-Cells in Atherosclerotic Plaques

### 3.1. CD3^+^ T-Cells

CD3 is a cell surface molecule that associates with the T-cell receptor (TCR) and is critical for the activation of CD4^+^ and CD8^+^ T-cells. It is commonly used as a pan T-cell marker in immunostaining protocols. In 1986, Jonasson et al. first demonstrated the presence of CD3^+^ T-cells in carotid plaques that were predominately located in the fibrous cap and constituted 20% of the total infiltrating cells [7]. A study of samples from autopsies revealed large numbers of CD3^+^ T-cells in the intimal and adventitial layers of coronary arteries displaying fatty streaks, with the highest numbers reported in advanced plaques with intimal thickening or necrotic cores [8]. A comprehensive histological study of human aortic tissues encompassing the entire spectrum of atherosclerotic disease confirmed the presence of CD3^+^ T-cells in the intima at all stages of atherosclerosis whereas CD3^+^ T-cells were present only in the medial layer of arteries displaying intimal thickening or in vulnerable plaques [9]. The authors also observed reduced numbers of CD3^+^ T-cells in the intima of healed ruptures and fibrotic calcified plaques [9]. Similarly, Rohm et al. reported higher numbers of CD3^+^ T cells in unstable plaques compared to stable plaques or vessels with no evidence of atherosclerosis [10].

Initial studies suggested that approximately 5–35% of CD3^+^ T-cells within plaques were activated and involved in the inflammatory response [11,12]. Immunofluorescent staining demonstrated increased expression of the chemokine CX3CL1 and its receptor CX3CR1 in the fibrous cap of coronary and carotid plaques [13]. The staining colocalized with CD3^+^ T-cells, suggestive of active recruitment of immune cells to inflamed tissues [13].

Previous studies using immunohistochemical or immunofluorescent staining investigated the location of immune cells within the plaque but do not reveal dynamic changes, assess the whole tissue, or provide detailed phenotypes of infiltrating cells. Flow cytometry allows unbiased quantitation and characterization of T-cells in affected tissues. For example, most cells isolated from endarterectomy specimens after collagenase digestion were found to be CD3^+^ T-cells, displayed a memory phenotype, and expressed markers of immune activation (e.g., CD25, HLA-DR) [12,14]. Combining flow cytometry and an ex vivo model of plaques, Lebedeva et al. confirmed the predominance of T-cells within carotid plaques [15].

### 3.2. CD4^+^ T-Cells

CD4^+^ T-cells have been shown to be more abundant in unstable plaques compared to stable plaques or arteries with no evidence of atherosclerosis [10]. Van Dijk et al. reported an absence of CD4^+^ T-cells in the intimal and medial layers of early plaques whereas their numbers were increased in late fibrous plaques but then decreased in healed plaque ruptures [9]. CD4^+^ T-cells with an effector memory or terminally differentiated phenotype have been found to predominate within plaques [16]. Additionally, intraplaque CD4^+^ T-cells displayed a more activated profile (i.e., increased HLA-DR expression) [16], suggesting the presence of foreign antigens within plaques. Using mass cytometry and single-cell RNA sequencing analyses, CD4^+^ T-cells in atherosclerotic plaques from symptomatic (stroke) patients displayed gene expression profiles consistent with activation, differentiation, and exhaustion whereas CD4^+^ T-cells were mostly activated in plaques from asymptomatic (no recent stroke) patients [17].

There is compelling evidence pointing to a role for Th1 CD4^+^ T-cells in promoting inflammation and atherosclerosis in humans. Using immunohistochemical and polymerase chain reaction techniques, Frostegard et al. demonstrated that Th1 CD4^+^ T-cells predominated in advanced plaques, whereas Th2 cytokines including IL-4 and IL-5 were rarely detected [18]. Other investigators confirmed the Th1 bias of infiltrating CD4^+^ T-cells in aortic or carotid plaques [19,20]. Intraplaque CD4^+^ T-cells have been shown to express the chemokine receptor CXCR3 and this was associated with expression of IFNγ-inducible CXC chemokines—IFN-inducible protein 10 (IP-10), monokine induced by IFNγ (MIG), and IFN-inducible T-cell α chemoattractant (I-TAC) in endothelial cells, smooth muscle cells, and macrophages within the plaques [21]. This study revealed a role for chemokine signaling through CXCR3 in promoting recruitment and homing of Th1 cells to the site of plaque development. Furthermore, the proinflammatory cytokines IL-12 and IL-18, which act synergistically to promote T-cell differentiation along the Th1 lineage, have been identified in atherosclerotic plaques [22,23,24]. IFNγ is known to activate macrophages and dendritic cells, inhibit proliferation of vascular smooth muscle cells, and reduce production of collagen by these cells, leading to thinning of the fibrous cap and plaque destabilization [25].

Although plaque-infiltrating CD4^+^ T-cells were shown to produce mainly IFNγ, other studies found some CD4^+^ T-cells produce only IL-17 or both cytokines following polyclonal stimulation [16,26]. This may explain the increased plasma levels of IL-17 from patients with acute myocardial infarction or unstable angina compared to patients with stable angina or healthy controls [27,28,29]. Furthermore, positive associations have been observed between IL-17 levels and severity of carotid artery plaques [30].

A subset of CD4^+^ T-cell lacking CD28 expression (representing 5–23% of all CD4^+^ T-cells) and producing high levels of IFNγ and tumor necrosis factor (TNF)α have been found in the plaques from patients with acute coronary syndrome (ACS) [31,32]. CD28 is a costimulatory receptor necessary for T-cell activation and proliferation, so the loss of CD28 has been suggested to reflect repeated antigenic exposure [33]. CD4^+^CD28^null^ T-cells have been shown to accumulate in unstable ruptured coronary plaque [31], and express high levels of OX40 and 4-1BB costimulatory proteins, which regulate degranulation and release of molecules involved in cytotoxicity including perforin and granzyme B [32]. These findings suggest that CD4^+^CD28^null^ T-cells may damage cells in the vascular wall, thereby affecting the stability of plaques.

### 3.3. CD8^+^ T-Cells

CD8^+^ T-cells have been found to be more abundant in unstable plaques compared to stable plaques [10]. CD8^+^ T-cells in advanced plaques resided mainly in the shoulder region and fibrous cap of the plaques [34]. Van Dijk et al. demonstrated CD8^+^ T-cells in the intimal layer throughout the progression of atherosclerosis, but these cells predominated in the medial layer of advanced unstable plaques and numbers decreased in stable lesions [9]. Plaques were found to be enriched for CD8^+^ T-cells that were primarily T_EM_ cells, displayed an activated phenotype, and expressed IFNγ, IL-2, or IL-17 [16]. Likewise, using mass cytometry and single-cell RNA sequencing analyses, Fernandez et al. reported plaque-derived CD8^+^ T-cells were predominantly differentiated and activated and displayed evidence of clonal expansion [17]. A recent study demonstrated an inverse correlation between percentages of CD8^+^ T-cells and macrophages in advanced plaques [35]. As this was not evident with CD4^+^ T-cells, the authors inferred that CD8^+^ T-cells may play a protective role by reducing macrophage accumulation [35]. Granzyme B has been shown to localize to CD8^+^ T-cells near the necrotic core of advanced plaques with a fibrous cap [36] and may be responsible for the apoptosis of macrophage-derived foam cells [37].

### 3.4. Treg Cells

Frequencies of FOXP3^+^ Treg cells have been shown to be higher in the intima of lipid-rich advanced plaques compared to early lesions [38]. Furthermore, Patel et al. found that increased number of FOXP3^+^ Treg cells and the Treg-associated cytokine IL-10 in carotid plaques were associated with symptomatic disease [39]. However, other investigators evaluating the plaque shoulder, which is prone to infiltrating immune cells, reported lower numbers of Treg cells in unstable plaques that correlated inversely with the number of dendritic cells [10,40]. Furthermore, unstable plaques were shown to have reduced mRNA expression of TGF-β [40], a Treg cell-associated cytokine that possess atheroprotective roles including suppression of lymphocyte and endothelial cell proliferation [41]. Results from experimental models have also identified several other potential mechanisms by which Treg cells and TGF-β production can affect plaque development and stability. These include inhibition of T- and B-cell effector functions, modulation of dendritic cell function and maturation, and inhibition of macrophage inflammation [42,43].

### 3.5. γδ T-Cells

γδ T-cells are important innate-like T-cells with a role in inflammation, infectious diseases, tumor surveillance, and autoimmunity. They represent 1–5% of circulating T-cells in the blood and recognize nonpeptide antigens such as lipids and phosphorylated nucleotides [44]. In apolipoprotein E knockout mice, γδ T-cells were found to be the major T-cell subset in early aortic root and arch lesions [45,46] and produced predominantly IL-17 [45]. Human studies of γδ T-cells in atherosclerotic lesions are limited. In humans, γδ T-cells have been demonstrated in plaques [47,48], with high frequencies observed in the aorta during the early stages (i.e., fatty streaks with infiltration of macrophages and lymphocytes but no foam cells) of atherosclerosis [47]. Further human studies are required to address the precise role of γδ T-cells in atherosclerosis.

### 3.6. NKT Cells

Natural killer T (NKT) cells are a unique subset of T-cells that express markers of NK and T-cells [49]. They can be classified as type 1 (invariant) or type 2 (CD1d-restricted) NKT cells. Invariant NKT cells express a restricted TCR repertoire comprising a Vα24-Jα18 TCRα chain that is preferentially paired with a Vβ11 TCRβ chain, whereas type 2 NKT cells have more variable TCRs. Invariant NKT cells recognize glycolipid antigens presented by the major histocompatibility complex (MHC) class I-like molecule CD1d expressed on antigen-presenting cells. Using flow cytometry, high percentages of NKT cells were identified in advanced plaques from patients with atherosclerosis [50]. Bobryshev et al. demonstrated NKT cells in the rupture-prone shoulders of advanced plaques that colocalized with CD1d-expressing dendritic cells [51]. Similarly, in plaques from patients with symptomatic atherosclerosis, Kyriakakis et al. revealed that up to 3% of infiltrating CD3^+^ T-cells stained for Vβ11 or Vα24 and confirmed the colocalization of NKT cells with CD1d^+^ cells [52]. Using immunohistochemical staining, an increased number of NKT cells were demonstrated in unstable plaques compared to stable lesions [10]. Plaque-derived NKT cells exhibited high sensitivity to antigen stimulation and also proangiogenic and pro-inflammatory activity, suggesting a role for these cells in plaque neovascularization and destabilization [52]. Furthermore, NKT cells isolated from atherosclerotic plaques in patients with abdominal aortic aneurysm promoted apoptosis of vascular smooth muscle cells via Fas, IFNγ production, and CD40 signaling which may affect stability of plaques [53].

## 4. B-Cells in Atherosclerotic Lesions

Compared to murine studies, evidence for a role of B-cells in human atherosclerosis is limited. In whole blood gene expression profiles from the Framingham Heart Study, genes associated with B-cell activation were reported to be expressed at higher levels in healthy controls compared to patients with coronary heart disease, suggesting a protective role [54]. However, B-cells have been demonstrated to be rare or undetectable by immunohistochemical staining [7,11] or constituted <1% of cells in plaques using flow cytometry [15]. Using polymerase chain reaction (PCR), Hamze et al. demonstrated the presence of IgG- and IgA-expressing B-cells in the arterial wall and showed that they produced proinflammatory cytokines including IL-6, TNF-α, and granulocyte-macrophage colony-stimulating factor (GM-CSF) [55].

## 5. Activation of T-Cells in Atherosclerotic Plaques

As mentioned previously, T-cells in atherosclerotic plaques mainly display an activated profile. Analysis of the T-cell repertoire in coronary plaques from patients with ACS or chronic stable angina revealed increased T-cells and specific T-cell clonotype expansions in unstable plaques but not in the peripheral blood, suggesting antigen-driven recruitment of T-cells to unstable plaques [56].

Several studies indicate that intraplaque T-cells are activated upon recognition of endogenous antigens. Heat shock proteins (HSP) are stress proteins that are conserved in prokaryotic and eukaryotic cells. Of these, HSP60 has been extensively studied in human atherosclerosis. Benagiano et al. found that in vivo activated CD4^+^ (but not CD8^+^) T-cells isolated from carotid plaques could be stimulated with human HSP60 to secrete IFNγ and TNFα [57]. Plaque-derived T-cells displayed increased reactivity against human HSP60 compared to peripheral blood-derived T-cells from the same individual and exhibited an oligoclonally restricted T-cell receptor repertoire, suggestive of antigen-driven proliferation within the plaque [58]. Furthermore, cocultures of HSP60-stimulated myeloid dendritic cells with T-cells isolated from plaques from the same individual were found to induce T-cell activation [59]. The authors also detected IFNγ, IL-6, TNFα, and IL-17 in myeloid dendritic cell–T-cell coculture supernatants and increased expression of the transcription factors T-bet (Th1) and RORγt (Th17) [59].

Production of reactive oxygen species and nitrogen species by endothelial cells mediates LDL oxidation in the vascular wall [60]. Scavenger receptors on macrophages have a high affinity for oxLDL resulting in lipid accumulation and the formation of foam cells. T-cell clones derived from plaques have been shown to become activated upon recognition of oxLDL [61]. Proliferation was observed when T-cells isolated from plaques were cultured with oxLDL-stimulated blood-derived dendritic cells [62,63]. This effect was abolished by atorvastatin (lipid-lowering medication) treatment [63] or reduced by inhibition of proprotein convertase subtilisin/kexin type 9 (PCSK9) [62].

Genetic studies have shown that a variant of bactericidal/permeability-increasing fold-containing family B member 4 (BPIFB4), a gene associated with longevity, may confer protection from cardiovascular diseases [64,65]. Mechanistically, Ciaglia et al. demonstrated that BPIFB4 promoted M2 polarization [66] and decreased activation of T-cells [67]. Further studies are warranted to determine whether BPIFB4 can be utilized as a novel therapeutic target in the management of atherosclerosis.

## 6. Role of Pathogens in the Pathogenesis of Atherosclerosis

Several pathogens have been implicated in the increased risk of cardiovascular disease. These pathogens, both bacteria and viruses, can infect several different cell types important in the pathogenesis of atherosclerosis, including monocytes and macrophages. Inflammation induced by these infectious agents may accelerate atherosclerotic plaque progression.

Cytomegalovirus (CMV) is a beta herpesvirus with a seroprevalence of 50–90% depending on age and geographic and socioeconomic status [68]. The association between CMV seropositivity and increased risk of cardiovascular disease is evident in epidemiological studies [69] of immunocompetent individuals, HIV-infected patients [70], and solid-organ transplant recipients [71].

Direct effects of CMV on atherosclerosis have been established in vivo by the detection of the virus in endothelial cells, smooth muscle cells, and monocytes/macrophages. CMV DNA has been identified in 63% of plaques obtained during coronary artery bypass graft surgery [72]. Compared to tissues with no evidence of atherosclerosis, CMV antigen and DNA were detected more frequently in atherosclerotic plaques from patients [73,74]. However, other investigators reported no differences in the detection of CMV DNA in atherosclerotic plaques compared to healthy vessels [75,76]. This may depend on the stage of atherosclerosis as one study identified CMV DNA in fatty streaks and early plaques, but it was rare in advanced plaques [77]. In contrast, another study reported higher CMV DNA and antigen in advanced plaques compared to early plaques [78]. CMV-positive cells were found to be mainly localized in the shoulder regions of the plaques but also in areas adjacent to the necrotic core and in the fibrous cap [79].

Nitiskaya et al. reported that 82% of plaques obtained from patients who underwent carotid endarterectomy had detectable CMV DNA and levels positively correlated with proportions of infiltrating T_EM_ CD4^+^ and CD8^+^ T-cells [80]. Similarly, Yaiw et al. demonstrated plaques positive for CMV had increased infiltration of CD3^+^ T-cells [79], suggesting that the virus may be involved in the inflammatory process. Indeed, in vitro studies showed that soluble factors (IFNγ, TNFα) secreted by CMV-specific T-cells can activate endothelial cells to produce chemokines (IP-10, fractalkine) and adhesion molecules that promote further recruitment of effector CD4^+^ and CD8^+^ T-cells [81,82] and induce apoptosis in activated endothelial cells [81].

Izadi et al. reported an association between the presence of CMV DNA in coronary atherosclerotic plaques with a history of unstable angina and myocardial infarction [83]. Furthermore, detection rate of CMV by immunohistochemistry in coronary plaques was higher in patients with ACS compared to patients with no ACS [84].

Epstein–Barr virus (EBV) is a common pathogen, with more than 90% of adults infected worldwide. EBV DNA has been detected in 17/30 plaques obtained during coronary artery bypass graft surgery [72]. Another study found EBV-specific cytotoxic T-cells and EBV DNA in 11/19 plaques from patients undergoing carotid endarterectomy [85]. The authors implicated EBV-specific T-cells in plaque inflammation as these cells were found throughout the intimal layer of the arteries, expressed the activation markers CD40L and CD25, and produced IFNγ and granzymes [85].

Epidemiological studies and randomized clinical trials have demonstrated an association between influenza infection and cardiovascular disease [86]. Influenza infection increased the risks of ACS and stroke, whereas vaccination against influenza reduced the risk of these major adverse cardiovascular events. Despite the absence of influenza A viral genome in atherosclerotic plaques, plaque-derived T-cells demonstrated high proliferative responses that were virus-specific and mainly involved CD4^+^ T-cells [87].

*Chlamydia pneumoniae* (*C. pneumoniae*) is a common pathogen in human respiratory tract infection and several studies have identified the bacteria in unstable plaques taken from patients who underwent carotid endarterectomy [88,89]. T-cell lines derived from carotid tissues have been found to proliferate in response to *C. pneumoniae* antigens, comprise predominantly CD4^+^ T-cells, and primarily display a Th1 cytokine profile [88]. Furthermore, the majority of the *C. pneumoniae*-specific cell lines were shown to be also responsive to HSP60 [88]. Symptomatic carotid plaques positive for *C. pneumoniae* demonstrated increased CD4^+^, CD8^+^, and CD45RO^+^ T-cells compared to asymptomatic plaques [90]. However, in symptomatic plaques without *C. pneumoniae*, CD4^+^ T-cells and CD45RO^+^ memory T-cells were increased, but not CD8^+^ T-cells when compared with asymptomatic plaques [90].

## 7. Clinical Utility of Profiling Circulating Cells of the Adaptive Immune System to Monitor Atherosclerosis

To date, there are no reliable biomarkers that can predict risk of major adverse cardiovascular events in individuals with subclinical atherosclerosis due to the difficulties in identifying unstable atherosclerotic plaques that are prone to rupture. Similar to noninvasive imaging modalities that are currently used to evaluate atherosclerotic plaque burden and composition, profiling T- and B-cells in blood specimens may provide vital information on disease progression. The development of multicolor flow cytometry technologies has facilitated detailed phenotypic and functional analyses of circulating immune cell subpopulations. Recent studies are summarized in Table 1.

### 7.1. CD4^+^ T-Cells

In multiple regression analyses, age, creatinine, and T_EM_ CD4^+^ T-cells were reported to be independent predictors of carotid intima–media thickness, a noninvasive measure of subclinical atherosclerosis [91]. The authors also observed increased frequencies of T_EM_ and activated CD4^+^ T-cells in patients with acute myocardial infarction or chronic stable angina compared with controls, whereas no differences were detected for CD4^+^ T-cells expressing the chemokine receptors, CCR5 or CXCR3 [91]. Increased percentages of highly differentiated CD4^+^ T-cells were found in ACS patients compared to patients with nonobstructive coronary artery disease (CAD) and healthy controls. Furthermore, higher degree of CD4^+^ T-cell differentiation correlated with more diseased vessels, lower left ventricular ejection fraction, increased number of prior ACS events, and worse SYNTAX score, which is an angiographic grading tool to determine the complexity of the CAD [92]. Together, these studies suggest ACS is associated with aging of the adaptive immune system and this correlates with measurements of disease pathology.

In patients with acute myocardial infarction or unstable angina, proportions of Th1 CD4^+^ T-cells were found to be higher compared to patients with stable angina with no differences observed for frequencies of Th2 and Th17 CD4^+^ T-cells between the two groups [93]. In a recent study, Li et al. reported increased percentages of Th1 CD4^+^ T-cells and Th1/Th2 ratio in patients with acute myocardial infarction compared to patients with stable CAD [94]. Proportions of Th1 T-cells and Th1/Th2 ratio also correlated with the number of affected coronary arteries, the degree of coronary artery stenosis, and lengths of lesions [94].

CD4^+^CD28^null^ T-cells have been shown to be increased in individuals with CAD compared to those without CAD [95]. Expression of the chemokine receptor CX3CR1 on CD4^+^CD28^null^ T-cells was also higher in patients with CAD than the control group [13] and this could explain the recruitment and accumulation of these cells within atherosclerotic plaques. Interestingly, CMV infection has been found to drive the accumulation of proatherogenic CD4^+^CD28^null^ T-cells in the circulation and these cells have the ability to recognize CMV antigens [109].

### 7.2. CD8^+^ T-Cells

Bergstrom et al. reported higher numbers and percentages of CD8^+^ T-cells expressing the natural killer cell marker CD56 in ACS and stable angina patients than in healthy controls independent of age, sex, and CMV seropositivity [96]. The authors also demonstrated higher proportions of CD8^+^CD56^+^ T-cells expressing IFNγ compared to CD8^+^CD56^−^ T-cells following stimulation, indicating a potential role for these cells in atherosclerosis progression and plaque instability [96]. In another study, Kolbus et al. investigated two subsets of activated CD8^+^ T-cells (CD8^+^CD25^+^ and CD8^+^CD56^−^ T-cells expressing IFNγ) as a predictor of acute myocardial infarction and ischemic stroke during 15-year follow-up [97]. They found that high frequencies of CD8^+^ T-cells were associated with increased incidence of coronary events but not ischemic stroke after adjustments for other cardiovascular risk factors [97]. Furthermore, proportions of CD8^+^CD25^+^ T-cells positively correlated with the degree of stenosis whereas inverse correlations were observed between the percentages of CD8^+^CD56^−^ T-cells expressing IFNγ and the degree of stenosis and carotid intima–media thickness, suggestive of differential roles of CD8^+^ T-cell populations in disease progression [97].

Frequencies of circulating CD8^+^ T-cells expressing CXCR3 have been reported to be increased in CAD patients [110]. These effector CD8^+^ T-cells can home to inflammatory sites via the chemokine IP-10, MIG, and I-TAC, which are expressed by endothelial cells, smooth muscle cells, and macrophages in human carotid atherosclerotic plaques [25]. Furthermore, production of IFNγ, perforin, and granzyme B by CD8^+^ T-cells can contribute to plaque rupture by promoting apoptosis of cells within lesions [111].

Percentages of T_EM_ CD8^+^ T-cells have been found to be increased in ACS patients compared to stable CAD patients [98] and in patients with CAD compared to healthy controls [112]. Detailed analyses of T_EM_ CD8^+^ T-cells revealed that they lacked the receptor for the proinflammatory cytokine IL-6 and possessed cytotoxic capabilities including the expression of perforin and granzyme B that can contribute to disease pathology [112]. Podolec et al. confirmed reduced frequencies of T_N_ CD8^+^ T-cells in patients with significant narrowing of the coronary arteries compared to individuals with no atherosclerosis [99]. Moreover, proportions of these cells correlated inversely with pulse wave velocity, a measure of arterial stiffness and a predictor of cardiovascular risk, in individuals without atherosclerosis, but not in patients with extensive coronary artery narrowing [99]. The authors suggested that monitoring of this cell subset may be useful during the early stages of coronary atherosclerosis.

Decreased circulating T_N_ CD8^+^ T-cells have also been reported in patients with ACS compared to individuals with stable CAD [99]. The authors showed that these cells displayed characteristics of immune exhaustion including impaired IL-12 production and upregulation of programmed cell death (PD)-1 molecule, and in vitro experiments indicated that oxLDL may contribute to this phenotype [99]. CD8^+^ T-cells that recognize and respond to oxLDL and HSP60 have been identified in patients with non-ST elevation myocardial infarction or stable angina but not in healthy controls [113]. This suggests that antigen-specific T-cells in atherosclerotic lesions can re-enter the circulation and therefore may be a useful indicator of plaque stability.

### 7.3. Treg Cells

The clinical utility of Treg cells as a metric of atherosclerosis progression has been complicated due to the different cell surface markers used to define these cells. Frequencies of Treg cells identified as CD4^+^CD25^high^CD127^low^ were found not to be associated with carotid intima–media thickness or progression of atherosclerosis [100]. In contrast, Hasib et al. demonstrated that naive but not memory Treg (defined using the markers CD4, FOXP3, and CD45RA) correlated inversely with right carotid intima–media thickness and also with the presence of atherosclerotic plaques [101].

Using FOXP3 and CD45RA, frequencies of resting and activated Treg cells were reported to be increased in patients with myocardial infarction or stable angina compared to individuals without cardiovascular disease [95]. However, several other investigators found reduced or no differences in the percentages of CD4^+^CD25^high^CD127^low^ and CD4^+^CD25^high^FOXP3^+^ Treg cells in patients with ACS compared to normal controls or patients with stable angina [93,101,102,103,104,105]. Peripheral blood cells from ACS patients cultured in vitro with simvastatin have been shown to increase the number and suppressive function of CD4^+^CD25^+^FOXP3^+^ Treg cells, suggesting a beneficial role for statins in stabilizing vulnerable atherosclerosis plaques [114].

### 7.4. B-Cells and NKT Cells

Frequencies of different B-cell subsets including CD19^+^ B-cells, unswitched (expressing IgM) and switched (expressing IgG or IgA) memory B-cells have been found to be reduced in patients who experienced a secondary cardiovascular event compared to those who did not [106]. A study of 700 individuals followed for 15 years in the Malmo Diet and Cancer Study showed lower baseline percentages of suppressive CD19^+^ B-cells expressing CD40 but higher activated CD19^+^ B-cells expressing CD86 in individuals with a later incidence of stroke [107]. However, these B-cell subsets were not associated with increased risk of CAD [107]. In patients undergoing percutaneous coronary intervention, proportions of iNKT cells were stable during acute myocardial infarction and follow-up [108].

## 8. Conclusions

**Figure 2:**
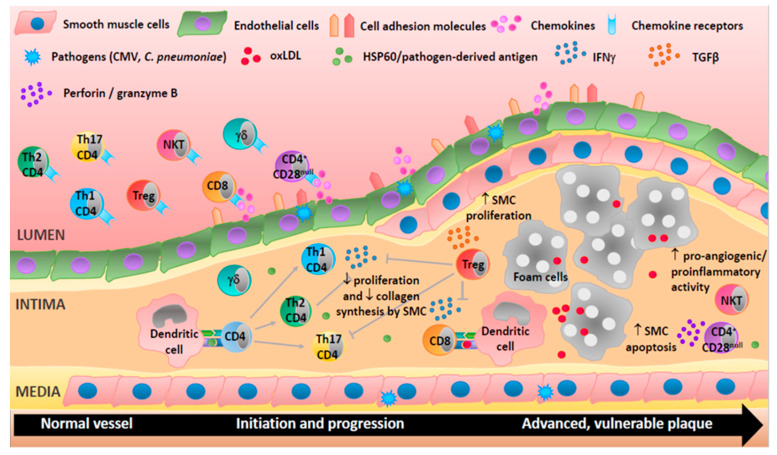
Adaptive immune cells are involved in all stages of human atherosclerosis. Endothelial cells activation upregulates cell adhesion molecules and secretion of chemokines and so directs T-cells to the site of inflammation. T helper (Th)1 cells produce interferon-γ (IFNγ), a proatherogenic cytokine able to activate macrophages, inhibit proliferation, and reduce collagen production by smooth muscle cells. Th2 cells produce interleukin (IL)-4 and may be atheroprotective as they can inhibit Th1 cells. CD4^+^CD28^null^ T-cells may damage cells in the vascular wall via the release of perforin and granzyme B. CD8^+^ T-cells may be proatherogenic via the production of IFNγ or protective by reducing macrophage content in the plaque. Treg cells can suppress Th1 and Th17 responses and increase smooth muscle cell proliferation through the secretion of cytokines (e.g., transforming growth factor (TGF)-β). Natural killer (NK)T cells exhibit proangiogenic and proinflammatory activities suggesting an involvement in plaque destabilization. Th17 and γδ T-cells are present in lesions but their roles are not well characterized. T-cells can be activated by heat shock proteins (e.g., HSP60), oxidized lipoproteins (oxLDL), or antigens derived from pathogens (e.g., cytomegalovirus (CMV) and *Chlamydia pneumoniae* (*C. pneumoniae*)).

Compelling evidence implicates adaptive immune cells in all phases of human atherosclerosis, from plaque initiation to destabilization (Figure 2). More extensive studies utilizing plaque tissues are necessary to characterize the effector cells and map critical pathways during atherogenesis. Studies in patients with different stages of atherosclerosis are now providing valuable insight, but heterogeneity of plaque characteristics hampers direct comparison between plaque features and circulating immune cells. Early detection of vulnerable lesions remains the main goal of biomarker discovery. Future studies including circulating cellular biomarkers are warranted to improve identification of vulnerable lesions so that effective intervention can be implemented before clinical manifestations are apparent.

## Figures and Tables

**Table 1:** Clinical utility of profiling circulating cells of the adaptive immune system to monitor atherosclerosis progression.

Immune Cell/s	Patient Group	Findings	Ref
CD4^+^CD28^null^	CAD, Controls	↑ frequencies of CD4^+^CD28^null^ T-cells expressing the CX3CR1 in patients with CAD compared to controls	[13]
CD4^+^ T-cells (Th1, Th17)	Acute MI (*n* = 26), UA (*n* = 16), SA (*n* = 16), Controls (*n* = 16)	↑ frequencies of Th1 and Th17 CD4^+^ T-cells in acute MI and UA patients compared with SA patients and healthy controls	[29]
NKT cells	Asymptomatic atherosclerosis patients (*n* = 10), Symptomatic atherosclerosis patients (*n* = 10), Controls (*n* = 10)	↓ frequencies of NKT cells in patients with atherosclerosis, with the lowest percentages reported in patients with symptomatic (defined as having a previous CV event) atherosclerosis	[52]
CD4^+^ T-cells	Chronic SAP (*n* = 30), Acute MI (*n* = 60), Controls (*n* = 40)	↑ frequencies of T_EM_ and HLA-DR CD4^+^ T-cells in chronic SAP and acute MI patients compared to controls Proportions of T_EM_ and HLA-DR CD4^+^ T-cells were similar in chronic SAP and acute MI patients Percentages of T_EM_ and HLA-DR CD4^+^ T-cells correlated with IMT	[91]
CD4^+^ T-cells, CD8^+^ T-cells, B-cells	Nonobstructive CAD (*n* = 21), ACS (*n* = 52), Controls (*n* = 50)	Absolute numbers of CD4^+^ T-cells and CD19^+^ B-cells similar in the three groups ↑ numbers of CD8^+^ T-cells in ACS patients compared to controls and nonobstructive CAD patients ↑ frequencies of highly differentiated CD4^+^ and CD8^+^ T-cells subsets in ACS patients Percentages of highly differentiated CD4^+^ and CD8^+^ T-cells were associated with worse SYNTAX score, greater number of affected vessels, lower LVEF, and increased number of prior ACS events	[92]
CD4^+^ T-cells (Th1, Th2, Th17, Tregs)	Acute MI (*n* = 19), UA (*n* = 25), SA (*n* = 20), Controls (*n* = 24)	↑ frequencies of Th1 cells in acute MI and UA patients compared to SA patients and controls Frequencies of Th17 and Th2 were similar in the four groups ↓ frequencies of Tregs (CD25^+^FOXP3^+^) in acute MI and UA patients compared with SA patients and controls	[93]
CD4^+^ T-cells (Th1, Th2)	Stable CAD (*n* = 35), STE (*n* = 30), NSTE (*n* = 35), Controls (*n* = 33)	↑ frequencies of Th1 cells and Th1/Th2 ratio in STE and NSTE patients compared to patients with stable CAD and controls Proportions of Th1 T-cells and Th1/Th2 ratio also correlated with the number of affected coronary arteries, the degree of coronary artery stenosis, and lengths of lesions	[94]
Tregs	CAD (SAP and previous MI) (*n* = 73), Controls (*n* = 64)	↓ frequencies of Tregs (FOXP3^+^) and T_reg_/T_eff_ ratio in CAD patients compared to controls ↑ expression of activation markers CD25 and CTLA4 on Tregs from CAD patients compared to controls T_reg_/T_eff_ ratio correlated inversely with levels of hs-HRP in CAD patients	[95]
CD8^+^ T-cells	SAP (*n* = 34), ACS (*n* = 30), Controls (*n* = 36)	↑ numbers and percentages of CD8^+^CD56^+^ T-cells were higher in ACS and SAP patients compared to controls	[96]
CD8^+^ T-cells	Subjects with a coronary event (*n* = 84), stroke (*n* = 54), or no event (*n* = 549) during the 15-year follow-up	High frequencies of CD8^+^ T-cells at baseline were associated with increased incidence of coronary events but not ischemic stroke High frequencies of CD8^+^CD56^-^ T-cells producing IFNγ at baseline were associated with increased incidence of ischemic stroke	[97]
CD8^+^ T-cells	Stable CAD (*n* = 66), ACS (*n* = 34)	↓ frequencies of T_N_ and ↑ frequencies of T_EM_ CD8^+^ T-cells in ACS patients compared to stable CAD patients	[98]
CD8^+^ T-cells	Patients with nonsignificant lesions (*n* = 41), Patients with severe lesions (*n* = 37), Controls (*n* = 36)	↓ frequencies of T_N_ CD8^+^ T-cells in patients with severe lesions than controls Percentages of T_N_ CD8^+^ T-cells correlated inversely with Gensini score Proportions of T_N_ CD8^+^ T-cells correlated inversely with PWV in controls but not in patients with atherosclerosis	[99]
Tregs	Chronic SAP (*n* = 36), NSTE ACS (*n* = 50), ST acute MI (*n* = 39), Controls (*n* = 75)	↓ frequencies of Tregs (CD25^high^CD127^low^) in non-ST ACS patients and ↑ frequencies of Tregs in ST acute MI patients compared to chronic SAP patients and controls	[100]
Tregs	ACS (*n* = 26), Post-ACS (*n* = 57, 24 STE, and 33 NSTE), Controls (*n* = 41)	↓ frequencies of naïve Tregs (FOXP3^+^ or CD25^high^CD127^low^) in ACS and post-ACS patients compared with controls Proportion of naïve Tregs correlated inversely with the presence of plaques on the right and left carotid and also with right carotid IMT	[101]
Tregs	ACS (*n* = 48), SAP (*n* = 24), Controls (*n* = 24)	↓ frequencies of Tregs (CD25^+^CD127^low^) in ACS patients compared with SAP patients and controls Frequencies of Tregs correlated inversely with levels of hs-CRP	[102]
Tregs	PCI with no disease progression (*n* = 32), PCI with new stenosis (*n* = 24), Patients with three-vessel coronary disease (*n* = 34), No atherosclerosis (*n* = 27)	↓ frequencies of Tregs (FOXP3^+^ or CD25^high^CD127^low^) in patients with multivessel atherosclerosis compared to individuals with no atherosclerosis Percentages of Tregs were similar in the three groups of patients Frequencies of Tregs correlated inversely with Gensini score in patients with multivessel atherosclerosis	[103]
Tregs	SAP (*n* = 34), ACS (*n* = 37), Controls (*n* = 35)	↓ frequencies of Tregs (CD25^+^CD127−) in ACS patients compared to SAP patients and controls Similar percentages of Tregs in SAP patients and controls	[104]
CD4^+^ T-cells, CD8^+^ T-cells, B-cells, Tregs	SAP (*n* = 13), ACS (*n* = 13)	No differences in percentages of CD4^+^ and CD8^+^ T-cells, activated (CD69^+^ or HLA-DR^+^) T-cells, B-cells, and Tregs (CD25^+^ and/or FOXP3^+^) between SAP and ACS patients	[105]
B-cells	Patients with advanced atherosclerosis who did not experience a secondary CV event during 3-year follow-up (*n* = 118) and those who did (*n* = 54)	↓ frequencies of CD19^+^ B-cells, unswitched (expressing IgM) and switched (expressing IgA or IgG) memory cells in patients who experienced a secondary CVD event compared to those who did not	[106]
B-cells	Individuals (*n* = 700) with a coronary event (*n* = 84), stroke (*n* = 66), or no event (*n* = 549) during 15-year follow-up	↓ proportions of suppressive CD19^+^CD40^+^ B-cells but ↑ frequencies of activated (CD19^+^CD86^+^) B-cells at baseline in individuals with a later incidence of stroke compared to those with no event B-cell subsets were not associated with increased risk of CAD	[107]
NKT cells	STE acute MI (PCI and follow-up) (*n* = 52)	Percentage and absolute number of NKT cells did not change during acute MI and at follow-up	[108]

Tregs, regulatory T-cells; T_EM_, effector memory T-cells; T_N_, naïve T-cells; ACS, acute coronary syndrome; LVEF, left ventricular ejection fraction; STE, ST elevation; NSTE, non-ST elevation; IMT, intima–media thickness; SAP, stable angina pectoris; UA, unstable angina; hs-CRP, high-sensitivity C-reactive protein; CAD, coronary artery disease; MI, myocardial infarction; PCI, percutaneous coronary intervention; CV, cardiovascular; CMV, cytomegalovirus; PBMC, peripheral blood mononuclear cells; PWV, pulse wave velocity; ↑, increased; ↓, decreased.

## Abbreviations

ACS	Acute coronary syndrome
BPIFB4	Bactericidal/permeability-increasing fold-containing family B member 4
CAD	Coronary artery disease
CMV	Cytomegalovirus
CV	Cardiovascular
DNA	Deoxyribonucleic acid
EBV	Epstein–Barr virus
FOXP3	Forkhead box 3
GM-CSF	Granulocyte-macrophage colony-stimulating factor
HIV	Human immunodeficiency virus
Hs-CRP	High-sensitivity C-reactive protein
HSP	Heat shock protein
IFNγ	Interferon gamma
IL	Interleukin
IMT	Intima–media thickness
IP-10	IFN-inducible protein 10
I-TAC	IFN-inducible T-cell α chemoattractant
LDL	Low-density lipoproteins
LVEF	Left ventricular ejection fraction
MHC	Major histocompatibility complex
MI	Myocardial infarction
MIG	Monokine induced by IFNγ
NKT	Natural killer T-cells
NSTE	Non-ST elevation
oxLDL	Oxidized low-density lipoproteins
PBMC	Peripheral blood mononuclear cells
PCI	Percutaneous coronary intervention
PCR	Polymerase chain reaction
PCSK9	Proprotein convertase subtilisin/kexin type 9
PD-1	Programmed cell death 1
PWV	Pulse wave velocity
RNA	Ribonucleic acid
SAP	Stable angina pectoris
STE	ST elevation
TCR	T-cell receptor
TGFβ	Transforming growth factor beta
Th	T helper
TNFα	Tumor necrosis factor alpha
Treg	Regulatory T-cells
T_CM_	Central memory T-cells
T_EM_	Effector memory T-cells
T_N_	Naïve T-cells
T_TE_	Terminal effector T-cells
UA	Unstable angina
